# Genetic Environment of *bla*_TEM-1_, *bla*_CTX-M-15_, *bla*_CMY-42_ and Characterization of Integrons of *Escherichia coli* Isolated From an Indian Urban Aquatic Environment

**DOI:** 10.3389/fmicb.2018.00382

**Published:** 2018-03-07

**Authors:** Nambram S. Singh, Neelja Singhal, Jugsharan S. Virdi

**Affiliations:** Microbial Pathogenicity Laboratory, Department of Microbiology, University of Delhi, New Delhi, India

**Keywords:** *Escherichia coli*, *bla*_CTX-M-15_, *bla*_CMY-42_, IS*26*, IS*Ecp1*, integrons, genetic environment, horizontal gene transfer

## Abstract

The presence of antibiotic resistance genes (ARGs) including those expressing ESBLs and AmpC-β-lactamases in *Escherichia coli* inhabiting the aquatic environments is a serious health problem. The situation is further complicated by the fact that ARGs can be easily transferred among bacterial species with the help of mobile genetic elements – plasmids, integrons, insertion sequences (IS), and transposons. Therefore, the analysis of genetic environment and mobile genetic elements associated with ARGs is important as these provide useful information about the epidemiology of these genes. In our previous study, we had reported presence of various β-lactam resistance genes present in *E. coli* strains inhabiting the river Yamuna traversing the National Capital Territory of Delhi (India). In the present study, we have analyzed the genetic environment of three ARGs *bla*_TEM-1_, *bla*_CTX-M-15_, and *bla*_CMY -42_ of those *E. coli* strains. The structure of class 1 integrons and their gene cassettes was also analyzed. Insertion sequence IS*26* was present upstream of *bla*_TEM-1_, IS*Ecp1* was present upstream of *bla*_CTXM-15_ gene and *orf477* was present downstream of *bla*_CTXM-15_. IS*Ecp1* was also present upstream of *bla*_CMY -42_ and, *blc* and *sugE* genes were present in the downstream region of this gene. Thus, the overall genetic environment surrounding these genes was similar to that reported from *E. coli* strains isolated globally. Conjugation assays, isolation and analysis of plasmid DNA of the transconjugants indicated that *bla*_TEM-1_, *bla*_CTX-M-15_, *bla*_CMY -42_ and class 1 integron were plasmid-mediated and possibly transmit between genera through horizontal gene transfer (HGT). This might lead to dissemination of antimicrobial resistance genes in aquatic environment. The work embodied in this paper is the first describing the genetic environment of *bla* and integrons in aquatic *E. coli* isolated from India.

## Introduction

Extensive use of third-generation cephalosporins for humans and veterinary purposes has led to an increased incidence and distribution of extended spectrum β-lactamases (ESBLs) and AmpC in bacteria (Bradford, 2001; Philippon et al., 2002; Bonnet, 2004; Jacoby, 2009). Antibiotic resistance in bacteria may emerge either due to genetic mutations in the antibiotic resistance genes (ARGs) present intrinsically or due to acquisition of foreign ARGs. The frequency of genetic mutations is normally low in nature (Zimmer et al., 1963; Gassmann et al., 2000), hence acquisition of ARGs through horizontal gene transfer (HGT) has been regarded as an important means for the wide spread antimicrobial resistance. It involves mobile genetic elements such as plasmids, transposons, and integrons.

The high prevalence of ESBLs and AmpC-β-lactamases in *Escherichia coli* is a world-wide public health concern, because *E. coli* is not only a common constituent of intestinal microbiota but also an important indicator of fecal contamination of aquatic environments. Most antibiotic-resistant *E. coli* strains enter the aquatic eco-system systems through various anthropogenic activities, discharge from livestock and poultry production, hospital and municipal wastewaters, etc. (Pruden et al., 2006; Pereira et al., 2013). Such waters when used for irrigation, drinking, or recreational activities disseminate antibiotic-resistant bacteria in the ecosystem (Pruden et al., 2006; Su et al., 2012; Pereira et al., 2013). The presence of antimicrobial resistance and their genes in *E. coli* in aquatic environments has been reported by many investigators (Koczura et al., 2013; Pereira et al., 2013). This is quite alarming, because such genes (ESBLs and AmpC) can be easily transferred among bacterial species with the help of mobile genetic elements, viz. plasmids, integrons, insertion sequences (ISs), and transposons (Liebana et al., 2013). Of these, integrons are of special concern because these are plasmid associated, hence can easily disseminate ARGs in bacterial species. These are very well-organized gene expression systems which can integrate one or several non-functional gene cassettes and convert these into functional genes (Recchia and Hall, 1995; Su et al., 2012). On the basis of the integrase gene (*intI*) integrons have been classified into three classes, class 1, 2, and 3 (Cambray et al., 2010; Su et al., 2012; Deng et al., 2015). IS elements have been closely associated with genes like *bla*_ESBLs_ and *ampC* and insertion sequence IS*Ecp1* helps in dissemination and expression of *bla*_CTX-M_ in *Enterobacteriaceae* (Poirel et al., 2006). Various investigators have described the genetic environment of *bla*_TEM_, *bla*_CTX-M-15_, and *bla*_CMY -42_ in clinical *E. coli* isolates. The genes were reportedly plasmid mediated and IS*26* was the most common IS element (Bailey et al., 2011; Dhanji et al., 2011; Hentschke et al., 2011; Mata et al., 2012). Also, IS*Ecp1* was reportedly associated with *bla*_CTX-M-15_ and *bla*_CMY -42_. *orf477* and *blc*-*sugE* were present downstream of *bla*_CTX-M-15_ and *bla*_CMY -42_, respectively (Bailey et al., 2011; Dhanji et al., 2011; Hentschke et al., 2011; Mata et al., 2012).

In our previous study (Bajaj et al., 2015b) we had reported various β-lactam resistance genes present in *E. coli* strains inhabiting the river Yamuna which traverses the National Capital Territory of Delhi (India). Of the ARGs, *bla*_TEM_ was the most widespread gene (100%), followed by *bla*_CTX-M-15_ (16%) and plasmid-mediated *ampC* (3%). Significant diversity was not observed in ESBLs as *bla*_CTX-M-15_ was the only ESBL detected. To the best of our knowledge, the genetic environment of *bla* has not been studied in *E. coli* isolated from the Indian aquatic environment. The analysis of genetic environment and mobile genetic elements associated with ARGs might provide useful information about the epidemiology of ARGs. Thus, the aim of the present study was to analyze the genetic environment associated with *bla*_TEM-1_, *bla*_CTX-M-15_, and *bla*_CMY -42_ in these *E. coli* strains. Detection of class 1 integrons and analyses of their gene cassettes was also carried out.

## Materials and Methods

### *E. coli* Isolates

A total of 61 well-characterized strains of *E. coli* belonging to four well-defined phylogroups (A, B1, B2, and D) isolated earlier from the river Yamuna and preserved at -80°C in 50% (v/v) glycerol were used in this study (Bajaj et al., 2015b). For subsequent experiments, the *E. coli* strains were revived in Luria-Bertani (LB) broth by overnight incubation at 37°C and 200 rpm. In the previous study from our laboratory, *bla*_TEM_ was reportedly present in all the 61 strains, *bla*_CTX-M-15_ in 10 and *amp*C (*bla*_CMY -42_) in only 2 strains (Bajaj et al., 2015b). The azide-resistant *E. coli* strain J53 which was used as the recipient during conjugation experiments was a kind gift from Dr. George A. Jacoby and provided to us by Dr. Sulagna Basu (National Institute of Cholera and Enteric Diseases, Kolkata, India).

### Characterization of Genetic Environment of *bla*_TEM-1_, *bla*_CTX-M-15_, *bla*_CMY -42_, Integrons, and Flanking Regions

DNA was extracted from *E. coli* strains by boiling lysis method (Rodríguez-Baño et al., 2004). The published primers (Lartigue et al., 2002) did not amplify the promoter region of *bla*_TEM_ in all the 61 *E. coli* strains studied; hence new primers were designed using an *E. coli* plasmid nucleotide sequence available at the GenBank (NCBI) as reference sequence (NC_010378.1). As insertion sequence IS*26* is reportedly present upstream of *bla*_TEM_ gene (Bailey et al., 2011), its presence and orientation relative to *bla*_TEM_ gene was investigated as described previously (Bailey et al., 2011; Ortega et al., 2012). The promoter and flanking regions of *bla*_CTX-M-15_ were successfully amplified using the published primers (Saladin et al., 2002; Dhanji et al., 2011) in all *bla*_CTX-M-15_ positive *E. coli* strains. The complete genetic environment of *bla*_CMY -42_ (*ampC*) was studied by overlapping PCR. The presence of genetic elements which frequently surround *ampC* was checked using the published primers (Pérez-Pérez and Hanson, 2002; Saladin et al., 2002). These were used to target the IS*Ecp*1 insertion sequence present upstream of the *bla*_CMY -42_. A newly designed primer set was used for amplifying the downstream region of *bla*_CMY -42_. The integrase genes *intI1, intI2*, and *intI3*, and integron class 1 gene cassette were detected using published primers (Kraft et al., 1986; Goldstein et al., 2001; White et al., 2001). It has been reported that the 3′-conserved segment (CS) which flank gene cassettes contain *qacE*Δ*1* and *sul1* genes which confer resistance to sulfafurazole antibiotics. Their presence was checked in nine strains which harbored class 1 variable gene cassette using primers and methods described previously (Guo et al., 2011).

The 25 μl PCR reaction mixture prepared contained 2.5 μl of 1× buffer, 200 μM of each dNTPs (Thermo Fisher Scientific, Waltham, MA, United States), 20 pmol of each forward and reverse primers, 6 μl of template DNA and 1 U of *Taq* DNA polymerase. The details of the primers and the PCR conditions are listed in **Table 1**. PCR amplicons were electrophoresed on 1% agarose gels at 80 V, stained with ethidium bromide and visualized using a UV tansilluminator. The PCR amplicons were purified using Hi-Yield^TM^ extraction kit (RBC Bioscience, New Taipei City, Taiwan) following manufacturer’s instructions and sequenced at a commercial facility using Sanger sequencing (Invitrogen BioServices India Pvt. Ltd., Bangalore, India). Homology search was performed for the nucleotide sequences using the BLAST algorithm available at NCBI^1^.

**Table 1:** Details of target genes, primers, annealing temperatures, and their amplicon size.

Primers	Nucleotide sequence	Target genes	Amplicon size (bp)	Annealing temperature (°C)	Reference
proF	5′-ATAAAATTCTTGAAGAC-3′	*bla*_TEM_	1069	42	Lartigue et al., 2002
proR	5′-TTACCAATGCTTAATCA-3′	Including promoter			
blaTEM full-f	5′-TAATAATGGTTTCTTAGACG-3′	*bla*_TEM_	1175	44	This study
blaTEM full-r	5′-CATGCATCTGTATAAGGGGT-3′	Including promoter			
IS26-f	5′-GCGGTAAATCGTGGAGTGAT-3′	IS*26, bla*_TEM_	Variable	55	Ortega et al., 2012
TEM1-r	5′-TCTTTTACTTTCACCAGCGTT-3′				
IS26a^+^-f	5′-ACCTTTGATGGTGGCGTAAG-3′	IS*26,bla*_TEM_	Variable	58	Bailey et al., 2011
TEM-r	5′-CCGGCTCCAGATTTATCAGC-3′				
IS26b^+^-f	5′-GATGCGTGCACTACGCAAAG-3′	IS*26, bla*_TEM_	Variable	58	Bailey et al., 2011
TEM-r	5′-CCGGCTCCAGATTTATCAGC-3′				
ISEcp1/U1	5′-AAAAATGATTGAAAGGTGGT-3′	IS*Ecp1, bla*_CTX-M-15_	900	48	Saladin et al., 2002
MA3	5′-ACYTTACTGGTRCTGCACAT-3′				
CTX-M	5′-CCGTTTCCGCTATTACAAAC-3′	*bla*_CTX-M-15_, *orf477*	1050	55	Dhanji et al., 2011
ORF477	5′-CTGGGACCTACGTGCGCCCG -3′				
ISEcp1-f	5′-AATACTACCTTGCTTTCTGA-3′	IS*Ecp1, bla*_CMY -42_	1831	60	Saladin et al., 2002
CMY-r	5′-CTGGGCCTCATCGTCAGTTA-3′				Pérez-Pérez and Hanson, 2002
Cmy-f	5′-CTTGAAAAGCTGCAATAACT-3′	*bla*_CMY -42_, *blc, sugE*	972	51	This study
SugE-r	5′-TCTGGAGCCTGATATGTCCT-3′				
Int1-F	5′-CCT CCC GCA CGA TGA TC-3′	*intI1*	280	60	Kraft et al., 1986
Int1-R	5′-TCC ACG CAT CGT CAG GC-3′				
hep58	5′-TCATGGCTTGTTATGACTGT-3′	Variable region	Variable	55	White et al., 2001
hep59	5′-GTAGGGCTTATTATGCACGC-3′				
qacE1-F	5′-AAGTAATCGCAACATCCG-3′	*qacE*Δ*1, sul1*	878	57	Bass et al., 1999
sul1-R	5′-GGGTTTCCGAGAAGGTGATTGC-3′				Nandi et al., 2004

*a^+^ and b^+^ The two possible orientations of IS*26* relative to *bla*_*TEM-1*_ (Bailey et al., 2011).*

### Conjugation and Analysis of Plasmid DNA

Conjugal transfer of plasmid-borne β-lactamase genes (*bla*_TEM-1_, *bla*_CTX-M-15_, *bla*_CMY -42_) and integrons was assessed by broth culture mating assay using *E. coli* J53 as recipient. After 24 h of incubation, mating mixtures of the donor and recipient were plated on agar containing sodium azide (100 μg/ml) and ampicillin (100 μg/ml) supplemented with either cefotaxime (8 μg/ml), or trimethoprim (10 μg/ml) or chloramphenicol (30 μg/ml). Plasmid DNA was extracted from the donors and transconjugants using a commercial kit (Plasmid Mini Kit, Qiagen GmbH, Hilden, Germany). The presence of the integrons and the resistance genes (*bla*_TEM-1_, *bla*_CTX-M-15_, *bla*_CMY -42_) associated with the plasmids was confirmed by PCR amplification of the plasmid DNA from transconjugant strains as template and analysis of the PCR amplicons after electrophoresis on 1% agarose gels.

### Accession Numbers

As the sequence of *bla*_TEM_ including its promoter region was identical in all the strains hence, the partial coding sequence (CDS) of only one representative strain (IP5N) was submitted to GenBank under the accession number: **MF576132**. Similarly, the partial CDS of IS*26* linked *bla*_TEM-1_gene of strains *E. coli* KK45 and *E. coli* KP24 were submitted to GenBank under the accession number **MF503681** and **MF503682**, respectively. The partial CDS of *bla*_CTX-M-15_ regions of 10 CTX-M-positive *E. coli* has been submitted under the accession numbers: **MF462194** and **MF477008–MF477016**. The accession numbers of the *bla*_CMY -42_ regions of AmpC-positive strains *E. coli* ISE and *E. coli* IPE were: **MF477017** and **MF462195**, respectively.

## Results and Discussion

### Genetic Environment of *bla*_TEM-1_

**FIGURE 1:**
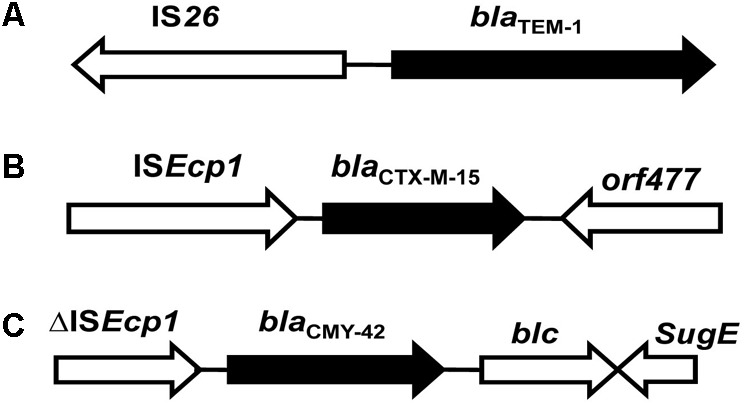
Schematic representation of genetic environment of *bla*_TEM-1_, *bla*_CTX-M-15_, and *bla*_cmy-42_ genes. The arrows indicate *bla* genes and upstream and downstream sequences detected by PCR. The direction of the arrows indicates the orientation of gene transcription. **(A**–**C)** Represents genetic environment of *bla_TEM-1_, bla*_CTX-M-15_, and *bla*_cmy-42_, respectively.

The genetic environment of *bla*_TEM-1_ is shown in **Figure 1A**. Analysis of the promoter regions of *bla*_TEM-1_ of all 61 strains revealed that promoters of *bla*_TEM-1_ of *E. coli* present in the river Yamuna were identical to the ‘*P3*-type promoter’ – the most commonly reported promoter associated with *bla*_TEM-1_ in *Enterobacteriaceae*. The typical regions at -10, TTCAAA and at -35, GACAAT were found to be 41 and 64 bp, respectively away from the starting codon. The *bla*_TEM-1_ gene of only three *E. coli* isolates, viz. KK45, IP24 and IST were linked to IS*26* insertion sequence in the upstream region, but at different positions. The orientation of IS*26* relative to *bla*_TEM-1_ in all the three Indian aquatic isolates was same as reported globally (**Figure 1A**; Bailey et al., 2011; Lin et al., 2014).

Expression of *bla*_TEM_ is associated with four different types of promoters, viz. *P3, Pa/Pb, P4*, and *P5* in the family *Enterobacteriaceae*, and *Haemophilus influenzae* (Lartigue et al., 2002; Tristram and Nichols, 2006; García-Cobos et al., 2008). Overexpression of *bla*_TEM-1_ has been associated mostly with *Pa/Pb* and *P4* type promoters. The Indian aquatic strains harbored the most commonly reported promoter upstream of *bla*_TEM-1_, i.e., *P3* type. Previous studies have reported more than 50% prevalence of *P3* promoters in TEM-1-positive, amoxicillin-clavulanate (AMC)-resistant *E. coli* clinical strains (Ortega et al., 2012). However, in our study no association was observed between AMC resistance and *P3*/TEM-1 in *E. coli* aquatic isolates as all the strains harbored *P3* type promoters irrespective of AMC resistance or sensitivity (Bajaj et al., 2015b). This suggested that the implications of the origin of *E. coli*, whether clinical or aquatic, on variations in promoter sequences and AMC resistance need to be investigated further.

Although *bla*_TEM-1_ was detected in all the 61 *E. coli* strains, insertion sequence IS*26* was linked with *bla*_TEM-1_ in only three strains, but at different positions. Many studies have reported the presence of insertion sequence IS*26* at different positions upstream of *bla*_TEM_ (Bailey et al., 2011; Ortega et al., 2012; Lin et al., 2014). It was observed that the *bla*_TEM_ genes preceded by IS*26* were also regulated by a *P3*-type promoter in these strains. Previous reports have shown that IS*26* can acquire two possible orientations relative to *bla*_TEM_ (Bailey et al., 2011). In the present study, it was observed that IS*26* acquired the most commonly reported orientation relative to *bla*_TEM_ (Bailey et al., 2011) as depicted in **Figure 1A**. It has been reported that the presence of a similar IS*26*-*bla*_TEM-1_ configuration in different species might indicate the geographical spread of a species, because IS*26* is often associated with transposons (Wain et al., 2003; Bailey et al., 2011). Researchers have so far not been able to understand the effect of IS*26* on the expression of *bla*_TEM_ (Ortega et al., 2012). IS*26*-*bla*_TEM-1_ configuration in Indian strains was similar to that reported for strains isolated from other parts of the world (Wain et al., 2003; Cain et al., 2010; Bailey et al., 2011).

### Genetic Environment of *bla*_CTX-M-15_

Investigation of the promoter regions of *bla*_CTX-M-15_ in all CTX-M-15-positive *E. coli* strains revealed the presence of insertion sequence IS*Ecp*1 containing typical -10 TACAAT and -35 TTGAA promoters region within its 3′ terminus, 48 bp away from the start codon. Analysis of the downstream region of the *bla*_CTX-M-15_ gene revealed presence of *orf477* which encodes for a hypothetical protein (**Figure 1B**).

Analysis of sequences flanking upstream and downstream of *bla*_CTX-M-15_ revealed the presence of IS*Ecp*1 upstream of all *bla*_CTX-M-15_-positive *E. coli* strains. The intact IS*Ecp1* was located 48 bp upstream of *bla*_CTX-M-15_ acquiring its preferential insertion sites which are usually located 42–266 bp upstream of different *bla*_CTX-M_ genes like CTX-M-1, CTX-M-2, and CTX-M-9 clusters. All the *bla*_CTX-M-15_-positive *E. coli* strains harbored the international *bla*_CTX-M-15_-type genetic environment. This organization has been reported previously in several *E. coli* strains isolated from France, Canada, Italy, United Kingdom, Spain, and China (Saladin et al., 2002; Boyd et al., 2004; Canton and Coque, 2006; Lavollay et al., 2006; Dhanji et al., 2011; Lin et al., 2014). The close association of *bla*_CTX-M_ and IS*Ecp1* is well known, and has been extensively reported from *E. coli* strains isolated from various geographical regions of the world highlighting the evolutionary association between IS*Ecp1* with *bla*_CTX-M_ (Karim et al., 2001; Saladin et al., 2002; Dhanji et al., 2011; Lin et al., 2014; Wang et al., 2014). IS*Ecp1* is a member of the family IS*1380* (IS Database home page^2^) and is weakly related to other IS elements (Lin et al., 2014). Zong et al. (2010) have reported the ability of IS*Ecp1* to mobilize an adjacent gene as a part of transposition units of different sizes. A single copy of IS*Ecp1* located upstream of *bla*_CTX-M_ successfully mobilized a chromosomal gene of a *Kluyvera* strain (Lartigue et al., 2006). It has also been reported that IS*Ecp*1 improves the expression of *bla*_CTX-M_, which is low in its natural source species but becomes high once acquired by a member of the family *Enterobacteriaceae* (Poirel et al., 2003). The intact IS*Ecp1* element contained the -10 TACAAT and -35 TTGAA promoter sequences within the 3′ non-coding region which were involved in transcription of *bla*_CTX-M-15_. IS*26* was reportedly associated with different variants of *bla*_CTX-M_ including *bla*_CTX-M-15_ in *E. coli* isolated from India and different parts of the world (Eckert et al., 2006; Ensor et al., 2006; Dhanji et al., 2011; Shahid et al., 2012; Lin et al., 2014; Wang et al., 2014). However, in our study, aquatic CTX-M-15-positive *E. coli* strains, unlike the clinical strains from India and abroad, did not show the presence of insertion sequence *IS26*. However, *orf*477 was found to be present in the downstream region of *bla*_CTX-M-15_, as reported earlier (Eckert et al., 2006; Dhanji et al., 2011; Wang et al., 2014).

### Genetic Environment of *bla*_CMY -42_ (*ampC*)

It was observed that *bla*_CMY -42_ genes harbored IS*Ecp*1 insertion sequence in the upstream and *blc* and *sugE* genes in the downstream region (**Figure 1C**).

Analysis of flanking regions of *bla*_CMY -42_ by overlapping PCR and sequencing revealed the presence of IS*Ecp1*, upstream of the gene (Hentschke et al., 2011; Mata et al., 2012; Tamang et al., 2012). However, the IS*Ecp*1 detected was found to be truncated at the 5′ end, as reported by several other researchers (Hentschke et al., 2011; Mata et al., 2012; Tamang et al., 2012). The genetic surroundings of *bla*_CMY -42_, in which the insertion sequence IS*Ecp1* has been reported to be disrupted by IS*1* (Hentschke et al., 2011) was not observed in any of the two CMY-42 positive aquatic *E. coli* strains. The genes encoding *blc* (outer-membrane protein) and *sugE* (drug-efflux channel) flanked the downstream region of *bla*_CMY -42_.

### Characterization of Class 1 Integrons and Flanking Sequences

Of the 61 *E. coli* strains, *intI1* was present in 30 strains. Nine of these strains harbored five different types of class 1 variable region gene cassette arrays which were present downstream of *intI1* and ranged between 0.9 and 2.8 kb in size (**Table 2**). The 5-gene cassette arrays contained a total of 18 gene cassettes, as follows: the dihydrofolate reductase (*dfr*) resistance gene family (*dfrA17, dfrA1, and dfrA7*), the aminoglycoside (*aad*) resistance gene family (*aadA5, aadA1, aadA2, aacA4*) and chloramphenicol (CHL) resistance gene *cat*B3. In seven of these strains, variable gene cassette arrays were found associated with *sul*1and *qacEΔ1* genes flanking the 3′-CS. The 3′-CS is known to contain *qacEΔ1* and *sul*1 genes which confer resistance to sulfafurazole antibiotics.

**Table 2:** Characteristics of class 1 integrons of Indian aquatic *E. coli* strains.

*E. coli* strains	Phylogroups	Class of integrase	*qacE*Δ*1* + *sul1*	Size of variable gene cassette array (bp)	Gene cassette arrays
IPG	A	*intI1*	+	2800	*aacA4, catB3, dfrA1*
NG28	A	*intI1*	+	1700	*dfrA1, aadA1*
IS5	A	*intI1*	-	-	-
KK36	A	*intI1*	-	-	-
NG9	A	*intI1*	-	-	-
MKNJ	A	*intI1*	-	-	-
WB23	A	*intI1*	-	-	-
KP5S	A	*intI1*	-	-	-
KP21	A	*intI1*	-	-	-
IST	A	*intI1*	-	-	-
PA21	A	*intI1*	-	-	-
WB28	A	*intI1*		-	-
IP24	B1	*intI1*	+	1900	*dhfr12, aadA2*
ISD	B1	*intI1*	-	-	-
NG29	B1	*intI1*	-	-	-
PA4	B1	*intI1*	-	-	-
MKNE	B1	*intI1*	-	-	-
SVI	B1	*intI1*	-	-	-
NG32	B1	*intI1*	-	-	-
IP5N	B1	*intI1*	-	-	-
WB14	B1	*intI1*	-	-	-
IPE	D	*intI1*	+	1700	*dfrA17, aadA5*
ISE	D	*intI1*	-	900	*dfrA7*
KK45	D	*intI1*	+	1700	*dfrA17, aadA5*
MKND	D	*intI1*	+	2800	*aacA4, catB3, dfrA1*
KK38	D	*intI1*	+	1700	*dfrA17, aadA5*
KK16	D	*intI1*	-	900	*dfrA7*
WB6	D	*intI1*	-	-	-
KK38	D	*intI1*	-	-	-
KKA	D	*intI1*	-	-	-
PA12	D	*intI1*	-	-	-

***+** Gene present, - gene absent.*

It has been reported that integrons carrying antimicrobial resistance gene cassettes were highly prevalent in aquatic environment (Guo et al., 2011; Canal et al., 2016). Moreover, Class 1 integrons have been reported widely in Gram-negative bacteria which were responsible for both the spread and increase of antimicrobial resistance throughout the world (Koczura et al., 2013; Canal et al., 2016). Our analysis also revealed that Class 1 integrons were common in *E. coli* isolates of river Yamuna (50%), more than those reported from strains isolated from Malaysia (21%) (Ghaderpour et al., 2015), France and Portugal (11%) (Laroche et al., 2009; Pereira et al., 2013), and Czechia (15%) (Dolejská et al., 2009). Class 2 and 3 integrons were absent in these strains. Class 3 integrons have also been reportedly absent in *E. coli* isolated from global aquatic habitats (Laroche et al., 2009; Su et al., 2012; Pereira et al., 2013). It would be pertinent to mention here that integrons of class 3 are rarely reported, even among *E. coli* isolated from humans and/or animals. In the current study, of the 30 integrase-positive isolates, variable regions were detected only in nine strains. This might be due to the presence of a cassette array that was too large to be amplified by the primers used as reported by other researchers also (Yu et al., 2003; Partridge et al., 2009; Ndi and Barton, 2011). Another reason might be the presence of a non-classic structure in the integron with the *tni* region or various ISs (Partridge et al., 2009).

The variable gene cassette regions of *E. coli* strains inhabiting the river Yamuna showed the presence of genes encoding for dihydrofolate reductase, *dfr* family (*dfrA17, dfrA1, dfrA7*), aminoglycoside adenyltransferase enzymes, the *aad* family (*aadA5, aadA1, aadA2, aacA4*) and chloramphenicol (CHL) resistance gene *catB3*. Since sulfonamide resistance gene (*sul1*) and quaternary ammonium compounds resistance genes (*qacEΔ1*) are often associated with the class 1 variable gene cassette region (Paulsen et al., 1993), their presence was also checked in the 3′-CS region of nine strains that harbored the variable gene cassette region. Of these, *sul1* and *qacEΔ1* genes were detected in seven isolates. Earlier reports in the literature indicated that *sul1* and *qacEΔ1* were not always present in the 3′-CS region of variable gene cassettes (Nass et al., 1998; Sáenz et al., 2004).

### Conjugation and Analysis of Transconjugants

Transferability of β-lactamase genes (*bla*_TEM-1_, *bla*_CTX-M-15_, *bla*_CMY -42_) and of class1 integron was checked by conjugation assay using all 61 *E. coli* strains as donor strains. Analysis of the plasmid DNA isolated from the recipients (transconjugants) revealed that *bla*_TEM-1_ present in all 61 strains, *bla*_CTXM-15_ in 10 strains, *bla*_CMY -42_ in 2 strains and the class 1 integron in 9 strains were plasmid mediated, and transferrable.

Conjugation assays indicated the transferability of resistance determinants (*bla*_TEM-1_ *bla*_CTX-M-15_, *bla*_CMY -42_, and class1 integrons) to a recipient strain of *E. coli* J53. Earlier studies have proved the transferability of resistant determinants like *bla*_CTX-M-15_, *bla*_TEM_, and plasmid-mediated quinolone resistance genes and integrons. These observations also indicated the possible transmission of these genes between genera through HGT (Guo et al., 2011; Haque et al., 2012; Bajaj et al., 2015a). It would be instructive to assess the enormity of such resistance gene transfer in the environment.

## Conclusion

The genetic environment of *bla*_TEM-1_, *bla*_CTX-M-15_, and *bla*_CMY -42_ in *E. coli* strains present in the urban aquatic environment of India has been reported for the first time. The overall genetic environment of β-lactamases was found to be similar to that reported from *E. coli* strains isolated globally. The Indian aquatic isolates harbored the most commonly reported *P3*-type promoter upstream of *bla*_TEM-1_. While *P3*/TEM-1 has reportedly been seen in AMC-resistant *E. coli* clinical strains worldwide, Indian aquatic isolates did not exhibit this organization. The *bla*_TEM-1_ was linked with IS*26* in its most commonly observed configuration as reported globally (**Figure 1A**). The Indian isolates harbored the international *bla*_CTX-M-15_-type genetic environment and IS*Ecp*1 was present upstream of *bla*_CTX-M-15_. Contrary to what has been reported for clinical strains isolated from India and elsewhere, aquatic *E. coli* strains lacked *IS26* element, but *orf*477 was present downstream of *bla*_CTX-M-15_. Class 2 and 3 integrons were absent, but class I integrons were found to be more common in *E. coli* isolates of the river Yamuna than that reported globally. The variable gene cassette regions revealed the presence of genes encoding for *dfr* family (*dfrA17, dfrA1, dfrA7*), the *aad* family (*aadA5, aadA1, aadA2, aacA4*), and *catB3*. The 3′-CS region showed the presence of *sul1* and *qacEΔ1* genes. The conjugation assays for class 1 integron and *bla*_TEM-1_, *bla*_CTXM-15_, and *bla*_CMY -42_ indicated simultaneous transfer of class 1 variable resistance gene cassettes and β-lactamases genes (*bla*_TEM-1_, *bla*_CTX-M-15_, *bla*_CMY -42_) implying that these were plasmid-mediated and possibly transmit between genera through HGT.

## Author Contributions

NSS and JV conceived and designed the experiments. NSS and NS did the data analysis. All the authors wrote the manuscript.

## Conflict of Interest Statement

The authors declare that the research was conducted in the absence of any commercial or financial relationships that could be construed as a potential conflict of interest.

## Footnotes

1 https://blast.ncbi.nlm.nih.gov/Blast.cgi

2 http://www-is.biotoul.fr
